# Comparison of unweighted and item response theory-based weighted sum scoring for the Nine-Questions Depression-Rating Scale in the Northern Thai Dialect

**DOI:** 10.1186/s12874-022-01744-0

**Published:** 2022-10-12

**Authors:** Suttipong Kawilapat, Benchalak Maneeton, Narong Maneeton, Sukon Prasitwattanaseree, Thoranin Kongsuk, Suwanna Arunpongpaisal, Jintana Leejongpermpoon, Supattra Sukhawaha, Patrinee Traisathit

**Affiliations:** 1 Department of Statistics, Faculty of Science, Chiang Mai University, 239 Huaykaew Road, Suthep, Muang, 50200 Chiang Mai, Thailand; 2 Department of Psychiatry, Faculty of Medicine, Chiang Mai University, Chiang Mai, Thailand; 3 Prasrimahabhodi Psychiatric Hospital, Ubon Ratchathani, Thailand; 4 Somdet Chaopraya Institute of Psychiatry, Bangkok, Thailand; 5 Department of Psychiatry, Faculty of Medicine, Khon Kaen University, Khon Kaen, Thailand; 6 Research Center in Bioresources for Agriculture, Industry and Medicine, Chiang Mai University, Chiang Mai, Thailand; 7 Department of Statistics, Faculty of Science, Data Science Research Center, Chiang Mai University, Chiang Mai, Thailand

**Keywords:** Depressive symptoms, Nine-Questions Depression-Rating Scale, Item response theory, Graded-response model, Nominal-response model, Differential item functioning

## Abstract

**Background:**

The Nine-Questions Depression-Rating Scale (9Q) has been developed as an alternative assessment tool for assessing the severity of depressive symptoms in Thai adults. The traditional unweighted sum scoring approach does not account for differences in the loadings of the items on the actual severity. Therefore, we developed an Item Response Theory (IRT)-based weighted sum scoring approach to provide a scoring method that is more precise than the unweighted sum score.

**Methods:**

Secondary data from a study on the criterion-related validity of the 9Q in the northern Thai dialect was used in this study. All participants were interviewed to obtain demographic data and screened/evaluated for major depressive disorder and the severity of the associated depressive symptoms, followed by diagnosis by a psychiatrist for major depressive disorder. IRT models were used to estimate the discrimination and threshold parameters. Differential item functioning (DIF) of responses to each item between males and females was compared using likelihood-ratio tests. The IRT-based weighed sum scores of the individual items are defined as the linear combination of individual response weighted with the discrimination and threshold parameters divided by the plausible maximum score based on the graded-response model (GRM) for the 9Q score (9Q-GRM) or the nominal-response model (NRM) for categorical combinations of the intensity and frequency of symptoms from the 9Q responses (9QSF-NRM). The performances of the two scoring procedures were compared using relative precision.

**Results:**

Of the 1,355 participants, 1,000 and 355 participants were randomly selected for the developmental and validation group for the IRT-based weighted scoring, respectively. the gender-related DIF were presented for items 2 and 5 for the 9Q-GRM, while most items (except for items 3 and 6) for the 9QSF-NRM, which could be used to separately estimate the parameters between genders. The 9Q-GRM model accounting for DIF had a higher precision (16.7%) than the unweighted sum-score approach.

**Discussion:**

Our findings suggest that weighted sum scoring with the IRT parameters can improve the scoring when using 9Q to measure the severity of the depressive symptoms in Thai adults. Accounting for DIF between the genders resulted in higher precision for IRT-based weighted scoring.

**Supplementary Information:**

The online version contains supplementary material available at 10.1186/s12874-022-01744-0.

## Background

Depression is a common mental disorder that is a leading cause of the global disease burden and deaths by suicide. In 2017, an estimated 264 million people (3.44%; range 2–6%) worldwide and 2.62 million people (3.09%) in Thailand experienced depression. The prevalence of depression in Thailand is slightly different between males and females (2.57% vs. 3.56%) and around twice higher in the elderly (50 years of age or more) than individuals aged 15–49 years old (6.02–6.29% vs. 3.37%) [1].

The measurement of psychological constructs such as depression and quality of life is complicated due to there being no way of assessing them directly. However, they can be quantified with an instrument, of which there are several for depression assessment, such as the Hamilton Rating Scale for Depression, the Beck Depression Inventory, the Montgomery-Åsberg Depression-Rating Scale, the Patient Health Questionnaire-9 (PHQ-9), the Calgary Depression Scale for Schizophrenia (CDSS), among others [2–6]. A Nine-Questions Depression-Rating Scale (9Q) in the northern Thai dialect is a measurement tool developed for assessing the severity of depressive symptoms in Thais in the northern region of the country since many people there do not use the formal Thai dialect in their daily lives, especially elderly people and those living in rural areas. Communication or interviewing involving technical terms in the formal Thai dialect could have led to misunderstanding. Researchers conducting a previous depression surveillance study in the northern region of Thailand using a two-question depression screening test (2Q) in the formal Thai dialect found that some participants denied the existence of symptoms related to depression due to the question not being relevant in their sociocultural context. Therefore, the 9Q in the northern Thai dialect was developed to reduce the possibility of misunderstanding due to the language barrier. It consists of nine rating scale items about the frequency and intensity of the diagnostic symptoms for major depressive disorder [7]. Scoring in the 9Q is commonly summed (ranging from 0 to 81 points) based on traditional techniques such as the Classical Test Theory (CTT). In contrast to the CTT approach, the Item Response Theory (IRT) is a technique for analyzing important aspects of measurements (e.g., item difficulty and item discrimination, as well as ordering of the response categories) and offers many advantages over CTT. The authors in [8] stated that an IRT model yields the estimated item and latent trait while taking variation according to the population characteristics into account, and thus can provide more comprehensive and accurate evaluations of item characteristics. Moreover, it can be applied to assess group differences for item and scale functioning and evaluate scales containing items with different response formats. In addition, it can also be helpful for developing better health outcome measures and for modeling changes over time. Moreover, it has been increasingly used as an alternative to CTT for measuring the development and validation of psychiatric disorders such as depression and anxiety [8–15]. The results from previous studies suggest that IRT approach may reveal additional information about the actual level of depression or other disorders compared to standard sum scoring [16–19].

Previously, researchers have suggested that IRT approach may reveal additional information about the actual level of depression or other symptoms compared to standard sum scoring [16]. Moreover, it may increase the precision in discriminating between individual differences in items over time [17]. The results of a simulation study indicate that the bias of estimating the rate of change over time was reduced by IRT-based scoring compared to standard sum scoring [20], possibly due to not assuming a constant error along the continuum of the measure, which is unlike CTT.

Previously, McNeish and Wolf [21] revealed that factor and IRT-based scoring are optimally weighted scales in which the loading for each item can be estimated differently. However, the sum-score approach is based on unit-weighting scoring that accounts for possible differences in the relationship between the latent trait score and each item, which can lead to less reliable scoring if the scales are scored differently. In addition, the authors also compared the results of using sum-scoring, factor-scoring, and simultaneous approaches on Verbal Cognition and Speeded Cognition for school membership. Their results showed that different scoring methods can result in different results; the first school membership group scored significantly higher on Verbal Cognition while the second group scored significantly higher on Speeded Cognition, which was different from the results using the factor-scoring regression and simultaneous approaches. This finding suggests that despite high correlations between the sum scores and factor scores (*R*^2^ = 0.97), small unexplained variances between the methods can lead to different conclusions. However, Widaman and Revelle [22] suggest that there was variation in factor loadings and factor scoring weights across the samples. Since the IRT approach takes the variation in population characteristics when estimating item parameters and latent traits into account [8], we hypothesized that applying IRT parameters as the weighted parameters for weighted sum scoring could be beneficial for mitigating this issue.

The PHQ-9 is commonly employed as a screening tool for depression and its severity in Thailand due to its excellent sensitivity and specificity for major depressive disorder [2]. However, considering only the frequency of symptoms might uncover the intensity of each symptom. Moreover, the standard sum score of PHQ-9 or 9Q based on CTT might lead to estimation bias between the demographics of the population and in the follow-up monitoring of people at risk over time. In addition, accounting for the differences of responses between genders when scoring for depression or depressive symptom severity has rarely been taken into account. Differential item functioning (DIF) is an approach to examine the difference in the probability of responding to an item among groups with the same psychological construct score. Previously, several researchers have found an impact of gender on the response pattern for a depression or depressive symptoms scale. In a study in Australia, researchers found that gender-related DIF was present in three symptoms associated with depression in the World Health Organization’s Composite International Diagnostic Interview [23]. The results of a study among Chilean adolescents indicate that DIF across gender was present in 6 of 13 items of the ASEBA School-Age Form Youth Self Report (YSR) used to measure depression and anxiety levels, among other disorders. These findings suggest that items found in commonly utilized measures for anxiety and depression symptoms may not represent the true level of behavioral problems unless DIF analysis is conducted based on gender [24]. The findings from another study on response patterns of Brazilian college students by using the Beck Depression Inventory-II (BDI-II) indicate that gender-related DIF was present in one item related to crying, implying that women are more likely to respond with a higher level of crying behavior than men even when they had a similar severity level of depression [25]. These studies reveal the importance of accounting for the difference in response patterns between genders. Therefore, our aim was to develop an IRT-based weighted sum scoring approach for a depressive symptom severity diagnosis tool that provides a more informative and precise indication of the actual levels of depressive symptoms as an alternative to the unweighted sum scoring approach by taking gender-related DIF into account. For that purpose, the performances of depressive symptom severity detection using the unweighted and IRT-based weighted scoring approaches for the 9Q were compared.

## Methods

### Settings and participants

We used secondary data from a study on the criterion-related validity of a revised 9Q in the northern Thai dialect comprising 1,527 individuals from the northern region of Thailand. This revised questionnaire was translated from the central Thai dialect version [7]. Participants who did not complete all items in the assessment or were under 19 years old were excluded from the study. The remaining participants were randomly stratified with proportional allocation for gender into two groups: a developmental group for IRT-based weighted scoring (*n* = 1,000) and a validation group for performance comparison.

### Assessments

The approach consisted of several parts, including demographics, screening for major depressive disorder, and diagnosis by an expert. All of the participants were first interviewed by a psychiatric nurse to obtain their demographic data and screen them for major depressive disorder using the revised two-question screening test [26]. They were then evaluated for depressive symptoms by a psychiatric nurse using the revised 9Q, which was blinded for another psychiatric nurse who evaluated them for major depressive disorder severity by using the Hamilton Rating Scale for Depression (HRSD–17). The participants were then interviewed by a psychiatrist to diagnose major depressive disorder based on the fourth edition of the American Psychiatric Association’s Diagnostic and Statistical Manual of Mental Disorders (DSM-IV) [27] and the MINI International Neuropsychiatric Interview-Thai version [28].

The 9Q was developed to assess the severity of depressive symptoms whereas the PHQ–9 was used to screen for depression. We hypothesized that considering only frequency of symptoms might not uncover the severity of depressive symptoms, and thus both the frequency and symptom intensity were accounted for in the product score in the calculation. Development of the 9Q in the northern Thai dialect included the following processes. (1) Psychiatrists and psychiatric nurses with experience of diagnosing depression and who spoke the northern Thai dialect consulted with experts in this dialect and patients/relatives living in northern Thailand to establish pertinent words and phrases for the questions about expressing feelings and mood in the formal Thai dialect version and the DSM-IV criteria by using the Delphi technique. (2) The study team formed a focus group involving the various populations in the northern area across age groups to ensure that the language used in this scale enabled efficient communication. (3) The developed tool was evaluated for construct validity and reliability by using exploratory factor analysis and Cronbach’s alpha coefficients, respectively.

The 9Q consists of nine rating scale items: (1) depressed mood (Mood); (2) markedly diminished interest or pleasure (Interest); (3) insomnia or hypersomnia (Sleep); (4) fatigue or loss of energy (Fatigue); (5) weight loss when not dieting or weight gain (Weight); (6) feeling of worthlessness or excessive or inappropriate guilt (Guilty); (7) diminished ability to think or concentrate, or indecisiveness (Concentration); (8) Psychomotor agitation or retardation (observable by others, not merely subjective feelings of restlessness or being slowed down) (Psychomotor); and (9) recurrent thoughts of death, recurrent suicidal ideation, or a suicide attempt or a specific plan for committing suicide (Suicide). The participant scored each item according to the perceived intensity (0 = no symptoms, 1 = mild, 2 = moderate, 3 = severe) and frequency (1 = several days, 2 = more than a week, 3 = nearly every day) of major depressive disorder symptoms within the previous two weeks. The score for each item was calculated as the product of the intensity and frequency scores. There are 7 plausible points for the product score of each item (0 = no symptoms, 1 = mild symptoms for several days, 2 = moderate symptoms for several days or mild symptoms for more than a week, 3 = severe symptoms for several days or mild symptoms nearly every day, 4 = moderate symptoms for more than a week, 6 = moderate symptoms nearly every day or severe symptoms for more than a week, and 9 = severe symptoms nearly every day). The total score for the 9Q ranges from 0 to 81 points. In the IRT procedure (i.e., assumption testing and parameter estimation), the 9Q product labels were defined as 0, 1, 2, 3, 4, 5, and 6 corresponding to the traditional 9Q scores of 0, 1, 2, 3, 4, 6, and 9, respectively.

### IRT models

This family of models can be used to measure an unobservable characteristic or a latent trait (*θ*) in individuals. An important difference between IRT and CTT is that the scale for the underlying latent variable that is being measured by a set of items is defined in IRT and the items are calibrated with respect to the scale. A commonly used IRT model for dichotomous items is the two-parameter logistic (2PL) model represented by two item parameters: item discrimination (*a*) and item difficulty (*b*).

Analogous to the 2PL model, IRT models for polytomous items (e.g., the Likert scale) have one discrimination parameter (*a*_*i*_) and a set of discrimination parameters for either the between-category threshold or the m-1 threshold (*b*_*ij*_) for each item. The discrimination parameter indicates the slope of the category response curves with a narrow and peaked curve indicating that the response category differentiates well across latent traits. The threshold parameters represent the location of the latent-trait level at which individuals have a 50% probability of endorsing the next category as an adjacent response category [29, 30]. The marginal maximum likelihood estimation (MMLE) using an expectation-maximization (EM) algorithm is suggested for parameter estimation [31, 32]. The polytomous IRT models used in our study were the graded-response model (GRM) (Eq. 1) and the nominal-response model (NRM) (Eq. 2):

1 $${\kern 1pt} {P_{ik}}(\theta )=\frac{{\exp \left[ {{a_i}\left( {\theta - {b_{ik}}} \right)} \right]}}{{1+\exp \left[ {{a_i}\left( {\theta - {b_{ik}}} \right)} \right]}} - \frac{{\exp \left[ {{a_i}\left( {\theta - {b_{i(k+1)}}} \right)} \right]}}{{1+\exp \left[ {{a_i}\left( {\theta - {b_{i(k+1)}}} \right)} \right]}},$$

2 $${\kern 1pt} {P_{ik}}(\theta )=\frac{{\exp \left[ {{a_{ik}}\theta +{c_{ik}}} \right]}}{{\sum\limits_{{k=1}}^{m} {\exp \left[ {{a_{ik}}\theta +{c_{ik}}} \right]} }},$$

where, ${\kern 1pt} {P_{ik}}(\theta )$ = The probability of responding to item *i* in category *k* (*k* = 0, 1, …, *m*).

*a*_*i*_ = A discrimination parameter for item *i*.

*a*_*ik*_ = A category slope parameter for item *i* in category *k*.

*b*_*ik*_ = A threshold parameter for item *i* in category *k*.

*c*_*ik*_ = A category intercept parameter for item *i* in category *k*.

Since the score for each 9Q item was calculated by multiplying its frequency and intensity, some of its values were equal even though their endorsements can be different. For example, the 9Q score of an individual who endorsed mild symptoms nearly every day (intensity = 1 multiplied by frequency = 3) is 3 points, which is the same as another individual who endorsed severe symptoms for several days (intensity = 3 multiplied by frequency = 1). Thus, there can be difficulties when accounting for this via the traditional ordering of the 9Q scores or nominal categorization using IRT-based scoring. Therefore, we applied the NRM for the categorical combination of symptom intensity and frequency on the nominal scale without natural ordering in addition to the GRM with ordering.

### Model selection

Prior to fitting the IRT model, the unidimensionality and local dependence assumptions were evaluated using a confirmatory factor analysis (CFA) with a maximum likelihood estimator, and local dependence was evaluated by using the residual correlation matrix resulting from the single factor CFA. Unidimensionality indices, including a comparative fit index (CFI) > 0.95, a Tucker Lewis index (TLI) > 0.95, and a root-mean-squared error of approximation (RMSEA) < 0.06, indicate that the fit of the model was adequate [33]. A residual correlation value of > 0.20 possibly indicates local dependence [34]. The monotonicity assumption was evaluated based on Loevinger’s *H* coefficient values for both the total scale (*H*) and each item (*H*_*i*_). The coefficients for the items (*H*_*i*_) of ≥ 0.30 and the total scale (*H*) of ≥ 0.50 proved that the monotonicity was acceptable [35, 36].

Likelihood-ratio testing was performed to compare the IRT models. The model with the lowest Akaike information criterion (AIC) and Bayesian information criterion (BIC) values was selected for model fitting [37].

The item-fit statistics of each item in the 9Q product and the 9QSF combination for the GRM were tested by using the chi2W method of Kondratek (2020) [38]. It is a Wald-type test statistic that compares the observed and expected item mean scores over a set of ability bins. It is available as a module in the Stata statistical software suite and can be used as an alternative method to assess the item-fit statistics for polytomous items.

### Differential item functioning (DIF)

This occurs when participants from different demographic groups (e.g., gender, age) with the same underlying trait score have a different probability of responding to an item. The presence of DIF may compromise comparisons across subgroups and can lead to misleading results, and measurement invariance cannot be presumed if DIF is present [39]. It can either be non-uniform, which is due to a statistically significant interaction between the trait level and the demographic variable (effect modification), or uniform, which is the difference between the strength of the relationship between the ability and the item responses in a model with and without the demographic variable for each item (confounding) [40].

An IRT-based technique was used to detect DIF for polytomous items. The baseline IRT models were fitted for all items and then compared to the other model with varied discrimination and threshold parameters between the reference and focal groups for each item. A comparison of models was performed using the likelihood-ratio test, with a significant difference (*p-*value) < 0.05 between the baseline and constrained model indicating the presence of DIF between the groups [39–41].

### IRT-based weighted scoring

The 9Q score (the sum-score of symptom intensity multiplied by the frequency of each item on an ordinal scale) and 9QSF (the categorical combination of symptom intensity and frequency on a nominal scale) was compared in this study. In the model selection procedure, GRM, which attained the lowest AIC and BIC values (Table 2) was used as the baseline model for IRT parameter estimation. For GRM, a discrimination parameter (*a*_*i*_) and threshold parameters (*b*_*ik*_) for *k* categories were estimated for each item *i*. However, GRM could not be used for parameter estimation using 9QSF due to the unordered scores of the categorical combinations. Thus, the IRT parameters for the 9Q score were estimated based on GRM while the parameters for 9QSF were estimated based on NRM with 10 plausible combined categories (0 = no symptoms, 11 = mild symptoms for several days, 12 = mild symptoms for more than a week, 13 = mild symptoms nearly every day, 21 = moderate symptoms for several days, 22 = moderate symptoms for more than a week, 23 = moderate symptoms nearly every day, 31 = severe symptoms for several days, 32 = severe symptoms for more than a week, and 33 = severe symptoms nearly every day). The number of each category combination was only used to label the category and was not based on the scoring. For the latter model, the *k*-1 category slope or category boundary discrimination (CBD) parameters for category *sf* (*a*_*i(sf)*_) and category intercept parameters for category *sf* (*c*_*i(sf)*_) were estimated for each item *i*.

We also tested IRT models without accounting for DIF (9Q-GRM and 9QSF-NRM) along with other models accounting for DIF (9Q-GRM-DIF and 9QSF-NRM-DIF). For example, we found that gender-related DIF was present in Item 2 and item 5 of the score under the GRM model. Therefore, the 9Q-GRM-DIF model was used to separately estimate threshold parameters for these items according to gender in the IRT-based weighted sum scoring.

For IRT-based weighted scoring, we considered that the threshold and discrimination parameters (based on the GRM) and the category slope parameters and category intercept parameters (based on the NRM) can be applied as the category weights and item weights for the weighted scoring for individual item scores. Thus, the IRT-based weighted sum score was calculated based on the weighted score for each item. The estimated values of the threshold parameters (*b*_*ik*_) under GRM were considered as the category weight for item *i* in category *k* whereas the estimated discrimination parameters (*a*_*i*_) were considered as the item weight for item *i*. The 9Q-GRM (or 9Q-GRM-DIF) score for individual *j* is defined as the linear combination of the product of the individual responses and the category weights weighted with item weights for all items divided by the plausible maximum of the product weighted score as follows:

3 $${\kern 1pt} 9{\text{Q-GR}}{{\text{M}}_j}{\text{ = }}\frac{{\sum\limits_{{i=1}}^{9} {\sum\limits_{{k=0}}^{6} {{a_i}{b_{ik}}{X_{ik}}} } }}{{\sum\limits_{{i=1}}^{9} {{a_i}{b_{i6}}{X_{i6}}} }},$$

where *a*_*i*_ is the discrimination parameter for item *i* (*i* = 1, 2, …, 9), *b*_*ik*_ is the threshold parameter for item *i* in category *k* (*k* = 0, 1, 2, 3, 4, 5, 6), and *X*_*ik*_ is the response of the individual for item *i* in category *k* (0 when category *k* is not endorsed or 1 when it is).

Meanwhile, under the NRM, combining the estimated category slope parameters (*a*_*i(sf)*_) and estimated category intercept parameters (*c*_*i(sf)*_) provides the category weights. The 9QSF-NRM (or 9QSF-NRM-DIF) score for individual *j* is defined as the linear combination of the individual weighted responses divided by the plausible maximum of the combined weighted score as follows:

6 $${\kern 1pt} 9{\text{QSF-NR}}{{\text{M}}_j}{\text{ = }}\frac{{\sum\limits_{{i=1}}^{n} {\sum\limits_{{sf=0}}^{{33}} {\left( {{a_{i(sf)}}+{c_{i(sf)}}} \right){X_{i(sf)}}} } }}{{\sum\limits_{{i=1}}^{n} {{\text{MAX}}\left( {\left( {{a_{i(sf)}}+{c_{i(sf)}}} \right){X_{i(sf)}}} \right)} }},$$

where *a*_*i(sf)*_ is the category slope parameter for item *i* (*i* = 1, 2, …, 9), *c*_*i(sf)*_ is the category intercept parameter for item *i* in category *sf* (*sf* = 0, 11, 12, 13, 21, 22, 23, 31, 32, or 33), and *X*_*ik*_ is the response of the individual for item *i* in category *k* (0 when category *k* is not endorsed or 1 when it is).

For example, under the GRM, assuming that the discrimination parameter of item 1 (mood) is 2.50 and the threshold parameters categorized from 0 to 6 are 0, 0.50, 1.00, 1.50, 2.00, 2.50, and 3.00, respectively, the item score is 7.50 (2.50 multiplied by 3.00) if the participant endorses a severe level for mood nearly every day. The sums of all of the item scores were calibrated on a 0–1 scale by dividing by the plausible maximum sum score, and the scale was then multiplied by 81 to enable comparison with the 9Q unweighted scores.

### Statistical analysis

The demographics of the participants are reported as frequencies and percentages for categorical variables and as medians and interquartile ranges (IQRs) for continuous variables. Differences between the demographic variable values of the developmental and validation groups were tested for significance by using Fisher’s exact test and the Mann-Whitney U test for categorical and continuous variables, respectively.

Differences between the means of the depressive symptoms severity levels using 9Q sum score (reference) were compared with 9Q frequency, 9Q-GRM, 9Q-GRM-DIF, 9QSF-NRM, and 9QSF-NRM-DIF by using analysis of variance (ANOVA) with Bonferroni adjustment. Pairwise comparisons for each category were compared using independent t-tests. The relative precision (RP) index was used to compare the performances of the two scoring procedures [42], the results of which are expressed as the ratio of the pairwise F-statistics (the IRT-based weighted score F-statistic divided by the unweighted sum-score F-statistic). This indicator is used to determine how much more or less precise the new scoring methods (9Q-GRM score, 9Q-GRM-DIF score, 9QSF-NRM score, and 9QSF-NRM-DIF score) are relative to the traditional method (9Q score) for distinguishing the severity of depressive symptoms. All analyses were performed using Stata version 17 (StataCorp, College Station, Texas 77,845 USA).

## Results

Of 1,527 individuals who participated in the primary study of the 9Q in the northern region of Thailand, 52 respondents (3.41%) who did not complete all of the items in the 9Q and the HRSD-17 were excluded from the analysis. Of the 1,355 participants aged 19 years old or more who were included in the study, 920 (67.90%) participants were female and the median age was 48 years old (IQR: 36–58). Most participants were married or living with a partner (64.99%). Two-hundred and fifteen participants (15.95%) were unemployed while around half of the participants (48.88%) had an income of less than 5000 baht/month. The major ethnicity and nationality of the participants were Thai (89.72% for ethnicity and 92.01% for nationality). Five hundred and twelve participants (38.18%) had at least one underlying disease (Table 1) such as hypertension, allergy, and/or diabetes mellitus. One thousand participants were randomly selected for the developmental group for the IRT-based weighted sum scoring while the remaining 355 participants were assigned to the validation group. There were no differences in the demographic characteristics between the two groups (Table 1).

**Table 1:** Characteristics of the participants (*N* = 1,355)

Demographic	All	Developmental Group	Validation Group	*p-*value
**(n (%) or Median [IQR])**	**(*N*** **= 1,355)**	**(*N*** **= 1,000)**	**(*N*** **= 355)**	
**Gender**				**0.523** ^**a**^
Male	435 (32.10%)	321 (32.10%)	114 (32.11%)	
Female	920 (67.90%)	679 (67.90%)	241 (67.89%)	
**Age**	**48 [36–58]**	**49 [36–58]**	**47 [36–57]**	**0.602** ^**b**^
19–59 years	1,086 (80.15%)	793 (79.30%)	293 (82.54%)	**0.108** ^**a**^
≥ 60 years	269 (19.85%)	207 (20.70%)	62 (17.46%)	
**Ethnicity** (***n*** **= 1,353)**				**0.889** ^**a**^
Thai	1,214 (89.72%)	897 (89.70%)	317 (89.80%)	
Thai-Yong	123 (9.09%)	92 (9.20%)	31 (8.78%)	
Thai-Laotian	4 (0.30%)	3 (0.10%)	1 (0.28%)	
Thai-Chinese	3 (0.22%)	2 (0.20%)	1 (0.28%)	
Tai Lue	7 (0.52%)	4 (0.40%)	3 (0.85%)	
Others	2 (0.15%)	2 (0.20%)	0 (0%)	
**Nationality (*n*** **= 1,352)**				**0.751** ^**a**^
Thai	1,244 (92.01%)	916 (91.88%)	328 (92.39%)	
Thai-Yong	104 (7.69%)	78 (7.82%)	26 (7.32%)	
Thai-Laotian	2 (0.15%)	1 (0.10%)	1 (0.28%)	
Tai Lue	2 (0.15%)	2 (0.20%)	0 (0%)	
**Relationship status (*n*** **= 1,354)**				**0.991** ^**a**^
Single	242 (17.87%)	179 (17.92%)	63 (17.75%)	
Married/with a partner	880 (64.99%)	650 (65.07%)	230 (64.79%)	
Divorced	100 (7.39%)	74 (7.41%)	26 (7.32%)	
Widowed	132 (9.75%)	96 (9.61%)	36 (10.14%)	
**Educational level (*n*** **= 1,345)**				**0.242** ^**a**^
None	25 (1.86%)	15 (1.51%)	10 (2.84%)	
Primary school	610 (45.36%)	459 (46.22%)	151 (42.90%)	
Lower secondary school	198 (14.72%)	145 (14.60%)	53 (15.06%)	
Upper secondary school	184 (13.68%)	136 (13.70%)	48 (13.64%)	
Diploma	173 (12.86%)	117 (11.78%)	56 (15.91%)	
Bachelor	139 (10.33%)	108 (10.88%)	31 (8.81%)	
Masters	16 (1.19%)	13 (1.31%)	3 (0.85%)	
**Occupation (*n*** **= 1,348)**				**0.223** ^**a**^
Unemployed	215 (15.95%)	161 (16.18%)	54 (15.30%)	
Employee	602 (44.66%)	451 (45.33%)	151 (42.78%)	
Government official	78 (5.79%)	61 (6.13%)	17 (4.82%)	
Merchant	149 (11.05%)	101 (10.15%)	48 (13.60%)	
Agriculturist	220 (16.32%)	164 (16.48%)	56 (15.86%)	
Business owner	52 (3.86%)	32 (3.22%)	20 (5.67%)	
Student	32 (2.37%)	25 (2.51%)	7 (1.98%)	
**Income (baht/month) (*n*** **= 1,340)**				**0.254** ^**a**^
< 5,000	655 (48.88%)	495 (49.95%)	160 (45.85%)	
5,000–10,000	434 (32.39%)	318 (32.09%)	116 (33.24%)	
10,001–20,000	198 (14.78%)	137 (13.82%)	61 (17.48%)	
20,001–40,000	39 (2.91%)	32 (3.23%)	7 (2.01%)	
40,001–60,000	9 (0.67%)	5 (0.50%)	4 (1.15%)	
> 60,000	5 (0.37%)	4 (0.40%)	1 (0.29%)	
**Underlying disease (*n*** **= 1,341)**				**0.278** ^**a**^
No	829 (61.82%)	620 (62.69%)	209 (59.38%)	
Yes	512 (38.18%)	369 (37.31%)	143 (40.63%)	

*N* Number of participants in each group, *n* Number of available observations, *IQR* Interquartile range

^a^ *p-*value derived from a Fisher’s exact test

^b^ *p-*value derived from a Mann-Whitney U test

**Fig. 1:**
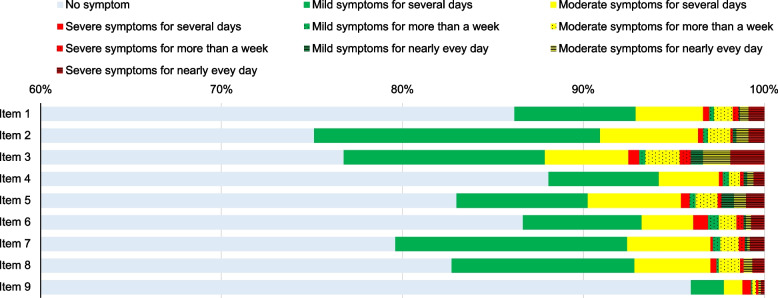
Item endorsements by the participants

According to item endorsement, more than 80% of the participants had no symptoms related to depression within the previous two weeks (except for items 2, 3, and 7). Item 3 had the highest endorsement rate of having severe symptoms nearly every day. Almost all of the participants (96%) did not report thoughts of physical self-harm or suicide (item 9) (Fig. 1).

The unidimensionality, local independence, and monotonicity assumption indices for the 9Q product and 9QSF combination used on participants aged 19 years old or over produced values close to the acceptance criteria. However, the values for participants aged 13–18 years old were poor (Supplementary Table 1). Therefore, IRT parameter estimation and scoring were only conducted on the participants aged 19 years old or over to avoid critical violations of the IRT assumptions. According to the model comparison using the likelihood-ratio test, GRM was the most appropriate model for all participants (AIC = 10710.43; BIC = 10898.05), as well as for males (AIC = 3299.51; BIC = 3442.14) and females (AIC = 7428.65; BIC = 7602.32). However, due to the unordered scores for categorical combinations, the NRM model was used to estimate the IRT parameters for the 9QSF even when its AIC and BIC values were a bit higher (Table 2). According to the item-fit statistics, 3 of the 9Q product items were a good fit for the GRM (Interest: χ^2^ = 1.75, *p* = 0.186; Guilt: χ^2^ = 1.37, *p* = 0.241; and Psychomotor: χ^2^ = 3.07, *p* = 0.080) whereas only one item from the 9QSF was suitable (Psychomotor; χ^2^ = 3.21, *p* = 0.073) (Table 3). The results of the DIF analysis show that there were significant differences in the responses to items 2 and 5 for the 9Q score and items 1, 2, 4, 5, 7, 8, and 9 for the 9QSF combination (Table 4). Therefore, we used both IRT models without accounting for DIF and the model accounting for DIF between males and females in this study.

**Table 2:** Item Response Theory model selection for the included participants aged ≥ 19 years (*N* = 1,355)

9Q Item Scoring	Group		GRM		NRM	
			**AIC**	**BIC**	**AIC**	**BIC**
**9Q frequency sum score**	**Participants**		10710.43	10898.05	10730.00	11011.43
	**Gender**	**Male**	3299.51	3442.14	3315.27	3523.17
		**Female**	7428.65	7602.32	7442.44	7702.95
**9Q unweighted sum score**	**Participants**		13162.36	13490.68	13213.99	13776.84
	**Gender**	**Male**	4012.09	4256.61	4031.50	4439.04
		**Female**	9163.92	9467.86	9211.65	9732.68
**9QSF combination**	**Participants**		13688.86	14147.48	13741.36	14559.58
	**Gender**	**Male**	4167.76	4473.41	4200.43	4734.30
		**Female**	9535.17	9950.06	9578.23	10311.54

*GRM* graded-response model, *NRM* Nominal-response model, *AIC* Akaike information criterion, *BIC* Bayesian information criterion, *NA* Not applicable

**Table 3:** Item-fit statistics for the 9Q product and 9QSF combination items’ suitability for the GRM by using chi2W item-fit statistics (adult participants; *N* = 1,355)

**9Q Product**	**9QSF Combination**
Mood (χ^2^=25.57, *p*<0.001)	Mood (χ^2^=39.37, *p*<0.001)
Interest (χ^2^=1.75, *p*=0.186)	Interest (χ^2^=6.48, *p*=0.011)
Sleep (χ^2^=7.57, *p*=0.006)	Sleep (χ^2^=9.28, *p*=0.002)
Fatigue (χ^2^=4.90, *p*=0.027)	Fatigue (χ^2^=4.49, *p*=0.034)
Weight (χ^2^=5.98, *p*=0.015)	Weight (χ^2^=10.46, *p*=0.001)
Guilt (χ^2^=1.37, *p*=0.241)	Guilt (χ^2^=7.07, *p*=0.008)
Concentration (χ^2^=4.76, *p*=0.029)	Concentration (χ^2^=9.96, *p*=0.002)
Psychomotor (χ^2^=3.07, *p*=0.080)	Psychomotor (χ^2^=3.21, *p*=0.073)
Suicide (χ^2^=22.79, *p*<0.001)	Suicide (χ^2^=45.38, *p*<0.001)

*p*-values were derived from the chi2W values for polytomous items according to Kondratek (2020) [38]

**Table 4:** DIF analysis between males and females (*N* = 1,355)

**Depressive Symptoms**	**9Q Score - GRM**		**9QSF Combination - NRM**	
	**NUDIF**	**UDIF**	**NUDIF**	**UDIF**
1. Depressed mood	0.574	0.464	<0.001^a^	<0.001^a^
2. Markedly diminished interest or pleasure	0.019^a^	0.012^a^	<0.001^a^	<0.001^a^
3. Insomnia or hypersomnia	0.166	0.213	0.137	0.106
4. Fatigue or loss of energy	0.557	0.414	<0.001^a^	<0.001^a^
5. Weight loss when not dieting or weight gain	0.006^a^	0.003^a^	0.009^a^	0.009^a^
6. Feeling of worthlessness or excessive or inappropriate guilt	0.085	0.059	0.333	0.341
7. Diminished ability to think or concentrate, or indecisiveness	0.454	0.388	<0.001^a^	<0.001^a^
8. Psychomotor agitation or retardation	0.115	0.072	<0.001^a^	<0.001^a^
9. Recurrent thoughts of death, recurrent suicidal ideation, or a suicide attempt or a specific plan for committing suicide	0.594	0.827	0.006^a^	0.001^a^

*9Q* The Nine-Questions Depression-Rating Scale, *GRM* Graded-response model, *NRM* Nominal-response model, *DIF* Differential item functioning, *NUDIF* Non-uniform differential item functioning, *UDIF* Uniform differential item functioning

^a^Significance of differential item functioning between males and females (*p*-value < 0.05)

The estimated IRT parameter values based on GRM for the 9Q score are reported in Table 5. For the GRM model accounting for DIF, the threshold parameters of items 2 and 5 were separately reported for males and females. Item 1 had the highest discrimination parameter values for both models while item 3 had the lowest. The IRT-based weighted sum score for the 9Q score was calculated by using the estimated discrimination parameters and the threshold parameters for items 1 through 9 for the validation group based on Eq. 5. The estimated IRT parameter values for the 9QSF combination based on NRM are reported in Table 6. Since endorsements for some combinations of the 9QSF were absent, we used the values from the other gender when they were absent for a particular gender or the values from the previous set of frequencies with the same intensity when they were absent for both genders. The category slope and intercept parameter values are reported separately for each category for the model without accounting for DIF and additionally separated by gender for the model accounting for DIF. The IRT-based weighted sum score of the 9QSF combination was calculated using the estimated parameters for the validation group based on Eq. 6. Examples of the raw score for each item, 9Q score, 9Q-GRM, 9Q-GRM-DIF, 9QSF-NRM, and 9QSF-NRM-DIF score are summarized in Supplementary Table 2.

**Table 5:** Estimated IRT parameter values for the 9Q score with the GRM for the developmental group

**Item**	**IRT Parameter Values Without Accounting for DIF**							**IRT Parameter Values Accounting for DIF**							
	***a***_***i***_	***b***_***i1***_	***b***_***i2***_	***b***_***i3***_	***b***_***i4***_	***b***_***i6***_	***b***_***i9***_	**Gender**	***a***_***i***_	***b***_***i1***_	***b***_***i2***_	***b***_***i3***_	***b***_***i4***_	***b***_***i6***_	***b***_***i9***_
Mood	4.043	1.111	1.510	1.926	1.998	2.263	2.534	All	3.834	1.189	1.581	1.986	2.056	2.311	2.570
Interest	3.182	0.766	1.464	2.059	2.183	2.504	2.758	M	2.160	1.055	1.796	2.705	2.806	2.920	3.467
								F	2.160	0.869	1.698	2.302	2.456	2.917	3.114
Sleep	1.284	1.137	1.894	2.475	2.655	2.997	3.525	All	1.361	1.173	1.894	2.446	2.618	2.943	3.443
Fatigue	3.004	1.303	1.774	2.303	2.436	2.670	2.990	All	3.072	1.360	1.820	2.333	2.578	2.929	3.115
Weight	1.425	1.461	2.013	2.702	2.972	3.351	3.776	M	2.160	1.259	1.675	1.932	2.113	2.442	2.805
								F	2.160	1.289	1.706	2.391	2.612	2.827	3.075
Guilty	2.191	1.399	1.876	2.320	2.513	2.820	3.187	All	2.265	1.446	1.909	2.341	2.527	2.823	3.176
Concentration	2.176	1.028	1.835	2.462	2.556	3.004	3.406	All	2.304	1.077	1.853	2.453	2.542	2.967	3.350
Psychomotor	2.063	1.202	1.830	2.443	2.535	2.912	3.376	All	2.194	1.242	1.843	2.427	2.513	2.871	3.311
Suicide	2.997	1.979	2.296	2.605	2.819	3.001	3.290	All	3.176	1.998	2.305	2.603	2.809	3.051	3.285

*a*_*i*_, Discrimination parameters for item *i*; *b*_*ik*_ Threshold parameters for item *i* in category *k*; *IRT* Item Response Theory, *GRM* Graded-response model, *DIF* Differential item functioning, *M* Male *F* Female

**Table 6:** Estimated IRT parameter values for the 9QSF combination with the NRM for the developmental group

**Item**	**Gender**	**IRT Parameters Values without Accounting for DIF**																	
		***a***_***i(11)***_	***a***_***i(12)***_	***a***_***i(13)***_	***a***_***i(21)***_	***a***_***i(22)***_	***a***_***i(23)***_	***a***_***i(31)***_	***a***_***i(32)***_	***a***_***i(33)***_	***c***_***i(11)***_	***c***_***i(12)***_	***c***_***i(13)***_	***c***_***i(21)***_	***c***_***i(22)***_	***c***_***i(23)***_	***c***_***i(31)***_	***c***_***i(32)***_	***c***_***i(33)***_
1	All	2.817	4.147	5.575	5.899	8.204	10.329	5.391	17.461	18.590	1.396	2.056	2.074	1.429	1.602	1.745	1.839	1.828	1.792
2	All	2.817	5.102	8.882	4.095	7.303	8.797	11.209	11.209^a^	12.589	0.896	1.713	1.710	1.180	1.499	1.630	1.730	1.730^a^	1.702
3	All	0.726	1.471	1.227	1.318	2.100	1.355	2.267	2.794	2.394	2.657	3.758	4.077	2.369	2.335	3.199	2.822	2.746	2.124
4	All	1.806	6.653	12.413	3.545	11.530	8.426	16.246	21.364	20.098	1.866	2.001	1.963	1.727	1.856	1.891	1.953	2.086	1.960
5	All	1.264	-0.987	2.110	1.584	1.757	1.573	1.237	1.879	2.199	2.214	-6.726	2.724	2.152	2.829	3.351	4.594	4.082	2.535
6	All	1.543	3.078	0.661	2.215	4.392	3.194	3.097	3.373	6.390	2.070	2.444	9.259	2.111	2.035	2.568	2.329	2.426	2.050
7	All	1.457	3.504	3.640	2.947	4.583	6.095	5.375	5.761	6.831	1.485	2.449	2.412	1.630	1.832	2.254	2.170	1.992	1.974
8	All	1.544	4.594	4.594^a^	2.213	3.027	4.682	3.270	2.832	5.722	1.711	2.335	2.335^a^	1.844	2.109	2.130	2.396	2.649	2.047
9	All	3.006	22.604	22.604^a^	3.784	5.533	8.547	7.059	5.426	7.316	2.164	2.644	2.644^a^	2.212	2.294	2.348	2.157	2.377	2.225
**Item**	**Gender**	**IRT Parameters Values Accounting for DIF**																	
		***a***_***i(11)***_	***a***_***i(12)***_	***a***_***i(13)***_	***a***_***i(21)***_	***a***_***i(22)***_	***a***_***i(23)***_	***a***_***i(31)***_	***a***_***i(32)***_	***a***_***i(33)***_	***c***_***i(11)***_	***c***_***i(12)***_	***c***_***i(13)***_	***c***_***i(21)***_	***c***_***i(22)***_	***c***_***i(23)***_	***c***_***i(31)***_	***c***_***i(32)***_	***c***_***i(33)***_
1	M	1.877	3.535^a^	4.823^a^	7.482	7.182	9.688	2.965	16.850	18.626	1.850	2.170^a^	2.177^a^	1.544	1.763	1.720	2.242	1.887	1.942
	F	1.877	3.535	4.823	4.416	7.203	6.588	8.015	10.973	12.933	1.680	2.170	2.177	1.498	1.636	2.001	1.854	1.845	1.751
2	M	1.877	5.161^a^	8.094^a^	3.785	6.309	5.527	11.454	11.454^a^	15.614	1.305	1.747	1.730^a^	1.354^a^	1.929	1.753	1.989	1.989^a^	2.071
	F	1.877	5.161	8.094	2.835	6.749	9.965	11.361	11.361^a^	11.784	1.014	1.747	1.730	1.376	1.536	1.746	1.774	1.774^a^	1.703
3	All	0.770	1.500	1.292	1.402	2.153	1.443	2.194	2.290	3.057	2.531	3.705	3.913	2.282	2.307	3.059	2.787	2.458	2.009
4	M	1.877	7.178	10.268^a^	4.539	10.076^a^	6.696	12.374^a^	17.574	10.240	1.941	2.006	1.921^a^	1.650	1.797^a^	1.851	1.895^a^	2.262	1.909
	F	1.877	6.386	10.268	3.231	10.076	9.269	12.374	17.574^a^	15.687	1.803	2.005	1.921	1.776	1.797	1.927	1.895	2.262^a^	1.943
5	M	1.877	-0.121	2.490	2.534	1.595	1.163	2.794	2.218^a^	2.045	1.758	-46.399	2.394	1.939	2.513	3.596	2.283	3.409^a^	2.399
	F	1.877	-4.018	2.619	1.651	3.144	3.148	-3.384	2.218	3.146	1.726	-3.104	2.392	1.955	2.292	2.454	-3.221	3.409	2.185
6	All	1.523	3.194	0.821	2.319	4.138	2.853	3.147	3.791	6.204	2.101	2.412	7.557	2.077	2.056	2.710	2.319	2.334	2.063
7	M	1.877	4.159^a^	4.698	5.225	5.786	6.666^a^	6.649	6.759	13.936	1.312	2.150^a^	1.856	1.469	1.815	2.085^a^	1.921	1.818	2.223
	F	1.877	4.159	4.698^a^	2.834	5.097	6.666	7.160	7.134	7.604	1.283	2.150	1.856^a^	1.603	1.708	2.085	2.077	1.923	1.865
8	M	1.877	4.798^a^	4.798^a^	2.925	1.882	7.366	9.494	6.674	10.526	1.440	2.200^a^	2.200^a^	1.560	2.912	1.744	1.996	1.967	1.972
	F	1.877	4.798	4.798^a^	2.384	3.708	7.366^a^	2.877	2.164	6.045	1.582	2.200	2.200^a^	1.796	1.920	1.744^a^	2.486	3.105	2.003
9	M	1.877	11.826^a^	11.826^a^	4.163	4.929^a^	9.665	9.195	4.550^a^	18.334	2.759	2.517^a^	2.517^a^	2.101	2.328^a^	2.181	2.062	2.454^a^	2.390
	F	1.877	11.826	11.826^a^	3.119	4.929	9.665^a^	5.990	4.550	3.777	2.684	2.517	2.517^a^	2.439	2.328	2.181^a^	2.337	2.454	2.591

*a*_*i(sf)*_ Category slope parameters for item *i* in category *sf*; *c*_*i(sf)*_ Category intercept parameters for item *i* in category *sf*; *IRT* Item Response Theory, *NRM* Nominal-response model, *DIF* Differential item functioning, *M* Male; *F* Female

^a^Replaced with the parameter value from the other gender when they were absent for a particular gender or the values from the previous set of frequencies with the same intensity when they were absent for both genders

Table 7 reports the mean and standard errors of the IRT-based weighted sum scores and unweighted sum scores for the validation group (*N* = 355). The IRT-based weighted sum scores were rescaled from 0 to 81 to directly compare them with the unweighted sum score (the 9Q score), after which it can be seen that the mean IRT-based weighted sum scores were higher than the 9Q unweighted score. Overall and pairwise comparisons between the means of the depressive symptoms severity groups show that they were significantly different. The RP values show that 9Q-GRM, 9Q-GRM-DIF, and 9QSF-NRM (1.140, 1.167, and 1.045, respectively) had higher precision than the unweighted sum scores. However, in the pairwise comparison, the RP values for IRT-GRM were lower than those for the 9Q score when comparing the mean values for no and severe depression. In addition, the RP of 9Q-GRM-DIF was higher than those of the other IRT models for almost all pairwise comparisons conducted in this analysis.

**Table 7:** Analysis of the depressive symptoms severity levels for the validation group (*N* = 355)

Scoring Model	Depressive Symptoms Severity				Statistic	Overall Comparison^a^	Pairwise Comparison^b^					
	None(*n* = 302)	Mild(*n* = 31)	Moderate(*n* = 14)	Severe(*n* = 8)								
	0	1	2	3			0 vs. 1	0 vs. 2	0 vs. 3	1 vs. 2	1 vs. 3	2 vs. 3
9Q unweighted sum score	1.37	6.52	15.76	32.25	Mean difference		5.15	14.39	30.88	9.24	25.73	16.49
(0–81 points)	(0.16)	(0.93)	(4.41)	(3.74)	F-statistic	164.82	35.64	133.24	356.25	80.35	190.88	237.65
(Reference)					RP	1.000	1.000	1.000	1.000	1.000	1.000	1.000
9Q frequency sum score	0.95	4.00	8.43	14.00	Mean difference		3.05	7.48	13.05	4.43	10	5.57
(0–27 points)	(0.10)	(0.43)	(1.55)	(1.55)	F-statistic	162.04	58.47	167.32	296.78	106.98	171.58	224.77
					RP	0.983	1.641	1.256	0.833	1.331	0.899	0.946
9Q-GRM score	3.00	14.33	30.63	48.09	Mean difference		11.33	27.63	45.09	16.3	33.76	17.46
(0–81 points)	(0.32)	(1.69)	(4.54)	(5.04)	F-statistic	187.95	73.92	209.17	324.53	134.11	192.09	258.34
					RP	1.140	2.074	1.570	0.911	1.669	1.006	1.087
9Q-GRM-DIF score	3.07	14.58	31.50	48.32	Mean difference		11.51	28.43	45.25	16.92	33.74	16.82
(0–81 points)	(0.32)	(1.67)	(4.59)	(4.79)	F-statistic	192.35	75.94	220.57	325.57	140.52	193.51	264.31
					RP	1.167	2.131	1.655	0.914	1.749	1.014	1.112
9QSF-NRM score	2.22	10.35	21.20	35.09	Mean difference		8.13	18.98	32.87	10.85	24.74	13.89
(0–81 points)	(0.22)	(1.29)	(3.51)	(4.51)	F-statistic	172.23	68.40	177.61	310.47	116.41	182.73	236.37
					RP	1.045	1.919	1.333	0.871	1.449	0.957	0.995
9QSF-NRM-DIF score	2.42	11.07	22.57	38.24	Mean difference		8.65	20.15	35.82	11.5	27.17	15.67
(0–81 points)	(0.25)	(1.35)	(3.99)	(4.94)	F-statistics	164.76	63.82	164.73	303.34	108.14	177.18	226.74
					RP	1.000	1.791	1.236	0.851	1.346	0.928	0.954

*n* frequency, *GRM* Graded-response model, *DIF* Differential item functioning, *RP* Relative precision

^a^ The overall comparison results obtained by using ANOVA with Bonferroni adjustment were significant for all of the models

^b^ The pairwise test results for the models using independent t-tests were significant

## Discussion

We conducted an observational study to develop an IRT-based weighed scoring approach for a depressive symptom assessment tool suitable for Thai adults. Individuals aged 19 years old or more from several areas of northern Thailand were interviewed, screened, and the severity of their depressive symptoms assessed by using the 9Q and HRSD-17, followed by a medical assessment. We discovered that using the IRT-GRM model while accounting for DIF for the 9Q score had a higher precision than the traditional unweighted sum score.

Several items with DIF attained a high discrimination parameter value for the actual depression trait. Although there are several measurement tools for depression and its severity suitable in different settings, ignoring differences in the discrimination parameter values of an item in a measurement tool can cause bias. Scoring of the discrimination and threshold parameters across characteristics (e.g., gender, underlying disease, etc.) based on the IRT approach might be useful for reducing bias in depression and severity measurements. According to the DIF analysis, we found that the responses to 9Q items 2 and 5 were different between males and females. This result, which is consistent with the findings from a previous study [43], could be due to the different underlying abilities of the gender groups or else different interpretations of the item responses. In addition to gender, it has also been reported that responses across age and ethnic groups are also sensitive to the DIF for some of the items in PHQ-9 [44, 45]. However, DIF analysis for between ethnic groups was not performed in this study due to an insufficient number of participants who were not Thai. Further study should be conducted to examine differences in responses for other characteristics of the participants not covered in this study.

Both NUDIF and UDIF according to gender were present in two items (item 2 “Markedly diminished interest or pleasure” and item 5 “Significant weight loss or gain”). The significant DIF values concerning depression could be due to the difference in the perception of or concern about psychological issues between the genders due to not only genetic but also social, biological, and environmental factors. According to the Thai culture, and especially in rural areas, women take care of the family and do housework whereas men work to earn money. However, men can relax with colleagues and/or friends more often than women. The differences in tasks, environment, and lifestyle could have led to women being more prone to diminished pleasure from life. The results from a previous study on patients undergoing treatment for painful conditions in an emergency department in the US indicate that the female patients presented higher scores for stress and anxiety than the male ones [46]. In this case, “interest” was the hallmark depressive symptom presenting a difference in responses between males and females, and so the evaluator would need to have been extra careful for this item when conducting the interview to prevent misdiagnosis and misinterpretation. In addition, the outcomes from a study on the impact of stressful life events on body mass index (BMI) changes also show that stressful life events are associated with an increase in BMI in females only [47]. The difference in this relationship might be due to DIF across gender groups.

When estimating the IRT parameters based on GRM for the 9Q score, we found that item 1 “Feeling down, depressed, or hopeless” had the highest discrimination parameter value, meaning that depressive symptoms are the most related to depression severity, a finding which is consistent with a previous study using CFA [48]. The results of the discrimination parameter analysis show differences in the correlation between depression severity and each item. Therefore, IRT procedures that can account for the different weights applied to the items seem to be appropriate for improving the scoring method for the 9Q adapted for northern Thais.

Our results show that accounting for DIF in the 9Q-GRM model provided higher precision (16.7%) than the traditional unweighted sum-score approach. This finding suggests that accounting for IRT discrimination and threshold parameters, as well as the differences between responses according to gender, could provide higher precision in 9Q scoring to evaluate the severity of the depressive symptoms. However, as the results of the 9QSF-NRM-DIF indicate, replacing the missing estimated parameters with previous categorical values when there are missing or non-responses for some of the plausible combination categories seems to be inappropriate. Recruiting more participants or finding alternative approaches (e.g., simulation) to complete the sample for all of the plausible 9QSF categories might improve the scoring precision.

Previously, the findings from a study using other depression severity measurement tools (the Patient Health Questionnaire (PHQ-9) and the Hospital Anxiety and Depression Scale (HADS)) also point toward age-related DIF for 3 PHQ-9 items (“little interest or pleasure in doing things”, “feeling down, depressed or hopeless”, and “feeling tired or having little energy”), which is consistent with the 9Q items with age-related DIF in our study [44]. However, the results from a recent study on the DIF of the PHQ-9 among healthcare workers in Thailand indicate that DIF was not found in items across age, gender, education, or alcohol consumption [49]. This suggests that DIF might be related to the none to low level of depression in the healthcare workers.

In addition, considering DIF for several factors could lead to estimating a large number of combinations of IRT parameters. The findings from a recent study on the impact of somatic symptoms on PHQ-9 scores suggest that although several items showed DIF with respect to disease-specific severity, salient DIF was present in the responses of very few patients [50]. Considering the impact of DIF on specific characteristics is worthy of further study.

There are limitations to this study, including no responses to some of the categories in the 9Q items, which makes it impossible to directly estimate the IRT parameters for several combinations of 9QSF combination models. Moreover, fewer participants had a moderate-to-severe level of depressive symptoms, which could have potentially caused estimation bias resulting in lower accuracy during parameter estimation involving these groups. In addition, we only performed the DIF analysis according to gender due to insufficient participants to create separate groups for other variables. Indeed, the parameter estimations might have been more precise when considering differences in responses according to characteristics other than gender. A further study with a larger sample size should be conducted to determine DIF in other variables and confirm the findings from the present study. Moreover, other approaches toward determining the DIF for polytomous items should be considered. Finally, the questionnaire used in this study was revised into the northern Thai dialect to interview only those Thais who understand it. The IRT parameters used in this scoring approach might be different when used in other settings. Finally, to prevent the necessity for psychiatrists, healthcare providers, or researchers to compile the IRT-based weighted sum score, we plan to develop a user-friendly website and/or smartphone app for practitioners to calculate the IRT-based weighted sum score automatically after inputting the raw data. However, accessing IT equipment and/or the Internet could be a limitation for its practical usage. Thus, modifying the IRT-based weighted sum scoring system to make the calculation easier under these circumstances would be useful.

## Conclusion

In summary, the findings for the parameters of the IRT models and scoring methods presented in this study suggest that we improved the scoring method for applying 9Q to measure the severity of depressive symptoms in Thai adults. Accounting for the DIF according to the gender of the participants resulted in higher precision both for overall and pairwise comparisons of mean depression scores using the IRT models. Our findings could improve the precision for evaluating depressive symptoms, which could lead to appropriate treatment according to the major depressive disorder severity.

## Supplementary Information

**Additional file 1:** **Supplementary Table 1. **Confirmatory factor analysis indices (*N* = 1,475). **Supplementary Table 2.** Raw scores and examples of scoring by using various major depressivedisorder assessment methods.

## Data Availability

The datasets used and/or analyzed during the current study are not publicly available due to lack of previous approval to share data publicly. The datasets used and/or analyzed during the current study can be made available through a data-sharing agreement with the corresponding author on reasonable request.
