# Supplementary material for: Comparison of unweighted and item response theory-based weighted sum scoring for the Nine-Questions Depression-Rating Scale in the Northern Thai Dialect
Source: BMC Med Res Methodol. 2022 Oct 12;22:268. doi: 10.1186/s12874-022-01744-0 (PMC9555165; doi:10.1186/s12874-022-01744-0)
Supplement: Supplementary file 1 — Additional file 1: Supplementary Table 1. Confirmatory factor analysis indices (N = 1,475). Supplementary Table 2. Raw scores and examples of scoring by using various major depressivedisorder assessment methods. [file 12874_2022_1744_MOESM1_ESM.docx]

**Supplementary Table 1.** Confirmatory factor analysis indices (N = 1,475).

| **IRT Assumption** | **Overall** | **Gender** | | **Age Range** | | | |
| --- | --- | --- | --- | --- | --- | --- | --- |
|  |  | **Male** | **Female** | **13–18** | **19–59** | **60+** | **19+** |
| **9Q product** |  |  |  |  |  |  |  |
| **Unidimensionality**^a^ |  |  |  |  |  |  |  |
| RMSEA | 0.078 | 0.099 | 0.094 | 0.168 | 0.092 | 0.120 | 0.084 |
| CFI | 0.953 | 0.931 | 0.932 | 0.554 | 0.935 | 0.925 | 0.949 |
| TLI | 0.937 | 0.908 | 0.909 | 0.405 | 0.913 | 0.900 | 0.932 |
| **Local independence** |  |  |  |  |  |  |  |
| Residual correlation range | 0.049 to 0.110 | -0.079 to 0.173 | -0.073 to 0.103 | -0.117 to 0.203 | -0.055 to 0.148 | -0.092 to 0.078 | -0.054 to 0.117 |
| Number of local dependence pairs (correlation >0.20) | 0 | 0 | 0 | 1 | 0 | 0 | 0 |
| **Monotonicity** |  |  |  |  |  |  |  |
| Total scale coefficient *H* | 0.448 | 0.431 | 0.462 | 0.224 | 0.440 | 0.564 | 0.462 |
| Item coefficient *H_i_* | 0.340–0.528 | 0.277–0.536 | 0.406–0.530 | 0.124–0.328 | 0308–0.522 | 0.447–0.636 | 0.347–0.540 |
| **9QSF combination (severity with frequency)** |  |  |  |  |  |  |  |
| **Unidimensionality**^a^ |  |  |  |  |  |  |  |
| RMSEA | 0.069 | 0.096 | 0.086 | 0.200 | 0.084 | 0.090 | 0.076 |
| CFI | 0.960 | 0.933 | 0.939 | 0.504 | 0.943 | 0.956 | 0.956 |
| TLI | 0.947 | 0.911 | 0.918 | 0.338 | 0.924 | 0941 | 0.941 |
| **Local independence** |  |  |  |  |  |  |  |
| Residual correlation >0.20 | -0.105 to 0.214 | -0.190 to 0.263 | -0.167 to 0.248 | -0.297 to 0.691 | -0.134 to 0.298 | -0.187 to 0.189 | -0.110 to 0.226 |
| Number of local dependence pairs (correlation >0.20) | 2 | 1 | 2 | 7 | 2 | 0 | 2 |
| **Monotonicity** |  |  |  |  |  |  |  |
| Total scale coefficient *H* | 0.439 | 0.437 | 0.445 | 0.222 | 0.434 | 0.554 | 0.455 |
| Item coefficient *H_i_* | 0.336–0.521 | 0.279–0.552 | 0.348–0.517 | 0.156–0.328 | 0.309–0.518 | 0.436–0.632 | 0.344–0.534 |

^a^ Confirmatory factor analysis with a maximum likelihood estimator was performed to derive the fit indices (RMSEA, root mean squared error of approximation; CFI, comparative fit index, TLI, Tucker-Lewis index).

9Q score = sum score of severity multiplied by the frequency of each item of 9Q; 9QSF = categorical combination of severity and frequency of 9Q

**Supplementary Table 2.** Raw scores and examples of scoring by using various major depressive disorder assessment methods.

| **ID** | **Item 1** | | **Item 2** | | **Item 3** | | **Item 4** | | **Item 5** | | **Item 6** | | **Item 7** | | **Item 8** | | **Item 9** | | **9Q** | **9Q-GRM** | **9Q-GRM-DIF** | **9QSF-NRM** | **9QSF-NRM-DIF** |
| --- | --- | --- | --- | --- | --- | --- | --- | --- | --- | --- | --- | --- | --- | --- | --- | --- | --- | --- | --- | --- | --- | --- | --- |
|  | **S** | **F** | **S** | **F** | **S** | **F** | **S** | **F** | **S** | **F** | **S** | **F** | **S** | **F** | **S** | **F** | **S** | **F** |  |  |  |  |  |
| **28** | 0 | - | 0 | - | 0 | - | 0 | - | 2 | 1 | 0 | - | 0 | - | 1 | 1 | 0 | - | 3 | 6.27 | 7.41 | 4.67 | 5.72 |
| **283** | 0 | - | 1 | 1 | 1 | 1 | 1 | 1 | 0 | - | 0 | - | 0 | - | 0 | - | 0 | - | 3 | 9.15 | 8.84 | 7.19 | 7.99 |
| **566** | 1 | 1 | 0 | - | 0 | - | 0 | - | 2 | 1 | 0 | - | 0 | - | 0 | - | 0 | - | 3 | 8.63 | 9.53 | 5.31 | 5.79 |
| **580** | 0 | - | 0 | - | 1 | 1 | 0 | - | 1 | 1 | 0 | - | 1 | 1 | 0 | - | 0 | - | 3 | 6.77 | 7.84 | 6.54 | 6.47 |
| **589** | 2 | 1 | 1 | 1 | 0 | - | 0 | - | 0 | - | 0 | - | 0 | - | 0 | - | 0 | - | 3 | 10.01 | 9.17 | 7.37 | 7.12 |
| **607** | 0 | - | 0 | - | 0 | - | 0 | - | 1 | 3 | 0 | - | 0 | - | 0 | - | 0 | - | 3 | 4.51 | 4.81 | 3.23 | 3.12 |
| **846** | 0 | - | 1 | 1 | 2 | 1 | 0 | - | 0 | - | 0 | - | 0 | - | 0 | - | 0 | - | 3 | 5.70 | 5.15 | 4.94 | 5.32 |
| **1101** | 0 | - | 1 | 1 | 0 | - | 0 | - | 0 | - | 1 | 1 | 1 | 1 | 0 | - | 0 | - | 3 | 9.07 | 8.82 | 6.85 | 7.83 |
| **1105** | 0 | - | 0 | - | 0 | - | 0 | - | 1 | 1 | 0 | - | 2 | 1 | 0 | - | 0 | - | 3 | 7.12 | 8.06 | 5.38 | 6.6 |
| **1112** | 0 | - | 1 | 1 | 0 | - | 0 | - | 1 | 1 | 0 | - | 0 | - | 1 | 1 | 0 | - | 3 | 8.2 | 8.54 | 6.97 | 8.05 |
| **1184** | 0 | - | 0 | - | 1 | 1 | 0 | - | 2 | 1 | 0 | - | 0 | - | 0 | - | 0 | - | 3 | 5.07 | 6.01 | 4.75 | 4.97 |
| **1514** | 0 | - | 0 | - | 0 | - | 0 | - | 0 | - | 0 | - | 2 | 1 | 1 | 1 | 0 | - | 3 | 7.58 | 8.06 | 5.23 | 6.40 |

GRM, graded-response model; DIF, differential item functioning; S, severity (0 = no symptoms, 1 = mild, 2 = moderate, 3 = severe); F, frequency (1 = several days, 2 = more than a week, 3 = nearly every day)

9Q score = sum score of severity multiplied by the frequency of each item of 9Q; 9QSF = categorical combination of severity and frequency of 9Q
